# Pushing the Efficiency of High Open‐Circuit Voltage Binary Organic Solar Cells by Vertical Morphology Tuning

**DOI:** 10.1002/advs.202200578

**Published:** 2022-03-21

**Authors:** Guilong Cai, Zeng Chen, Xinxin Xia, Yuhao Li, Jiayu Wang, Heng Liu, PingPing Sun, Chao Li, Ruijie Ma, Yaoqiang Zhou, Weijie Chi, Jianqi Zhang, Haiming Zhu, Jianbin Xu, He Yan, Xiaowei Zhan, Xinhui Lu

**Affiliations:** ^1^ Department of Physics The Chinese University of Hong Kong New Territories Hong Kong 999077 China; ^2^ State Key Laboratory of Modern Optical Instrumentation Center for Chemistry of High‐Performance & Novel Materials Department of Chemistry Zhejiang University Hangzhou Zhejiang 310030 China; ^3^ School of Materials Science and Engineering Peking University Beijing 100871 China; ^4^ School of Mechanical and Aerospace Engineering Nanyang Technological University Singapore 639798 Singapore; ^5^ Department of Chemistry and Hong Kong Branch of Chinese National Engineering Research, Center for Tissue Restoration & Reconstruction Hong Kong University of Science and Technology (HKUST) Clear Water Bay Hong Kong 999077 China; ^6^ Department of Electronic Engineering The Chinese University of Hong Kong New Territories Hong Kong 999077 China; ^7^ Fluorescence Research Group Singapore University of Technology and Design Singapore 487372 Singapore; ^8^ CAS Key Laboratory of Nanosystem and Hierarchical Fabrication CAS Center for Excellence in Nanoscience National Center for Nanoscience and Technology Beijing 100190 China

**Keywords:** open‐circuit voltage, organic solar cells, power conversion efficiency, solid additive, vertical phase separation

## Abstract

The tuning of vertical morphology is critical and challenging for organic solar cells (OSCs). In this work, a high open‐circuit voltage (*V*_OC_) binary D18‐Cl/L8‐BO system is attained while maintaining the high short‐circuit current (*J*_SC_) and fill factor (FF) by employing 1,4‐diiodobenzene (DIB), a volatile solid additive. It is suggested that DIB can act as a linker between donor or/and acceptor molecules, which significantly modifies the active layer morphology. The overall crystalline packing of the donor and acceptor is enhanced, and the vertical domain sizes of phase separation are significantly decreased. All these morphological changes contribute to exciton dissociation, charge transport, and collection. Therefore, the best‐performing device exhibits an efficiency of 18.7% with a *V*_OC_ of 0.922 V, a *J*_SC_ of 26.6 mA cm^−2^, and an FF of 75.6%. As far as it is known, the *V*_OC_ achieved here is by far the highest among the reported OSCs with efficiencies over 17%. This work demonstrates the high competence of solid additives with two iodine atoms to tune the morphology, particularly in the vertical direction, which can become a promising direction for future optimization of OSCs.

## Graphical abstract

The solid additive, 1,4‐diiodobenzene (DIB), has a high competence to optimize the vertical morphology of bulk heterojunction active layer, resulting in a high efficiency of 18.7% with a open‐circuit voltage *(V*_OC_) of 0.922 V, a short‐circuit current *(J*_SC_) of 26.6 mA cm^−2^, and a fill factor (FF) of 75.6% for DIB‐processed D18‐Cl/L8‐BO binary devices.

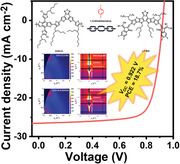

## 1 Introduction

Compared with conventional solar cells, bulk heterojunction (BHJ) organic solar cells (OSCs) have attracted considerable attention by virtue of their potential to deliver low‐cost, solution‐processable, flexible, and environment‐friendly solar cell devices.^[^ ^1^, ^2^, ^3^, ^4^ ^]^ Along with the evermore in‐depth understandings of material design strategies,^[^ ^5^, ^6^, ^7^, ^8^, ^9^, ^10^, ^11^, ^12^, ^13^, ^14^, ^15^ ^]^ device preparation procedures,^[^ ^16^, ^17^, ^18^, ^19^, ^20^ ^]^ morphological^[^ ^21^, ^22^, ^23^, ^24^ ^]^ and physical^[^ ^25^, ^26^, ^27^, ^28^, ^29^, ^30^ ^]^ mechanisms, the power conversion efficiencies (PCEs) of OSCs have been improved progressively in recent years. In particular, the syntheses of milestone nonfullerene acceptors ITIC^[^ ^8^ ^]^ and Y6^[^ ^10^ ^]^ have dramatically boosted the PCEs of single‐junction OSCs to exceed 18%.^[^ ^12^, ^31^, ^32^, ^33^, ^34^, ^35^, ^36^, ^37^, ^38^, ^39^, ^40^ ^]^ However, to date, most of the high‐performance single‐junction OSCs with PCEs of >17% have open‐circuit voltages (*V*_OC_) lower than 0.9 V, significantly deviating from the condition to achieve the theoretical PCE limit of OSCs. Therefore, extensive research efforts have been devoted to elevating the *V*_OC_ while maintaining the high short‐circuit current density (*J*_SC_) and fill factor (FF) of single‐junction OSCs, in expectation of pushing the PCE toward 20%.

For a binary system, the theoretical *V*_OC_ limit is set by the difference between the highest occupied molecular orbital (HOMO) of the donor (D) and the lowest unoccupied molecular orbital (LUMO) of the acceptor (A), which is usually realized by broadening the bandgap of donor and/or acceptor while minimizing their LUMO offset as long as there is a sufficient driving force for exciton dissociation. However, the bandgap increase is at the cost of absorption band width, which makes it challenging to achieve high *V*_OC_ and *J*_SC_ simultaneously. Nevertheless, practically obtained *V*_OC_ and *J*_SC_ are still far from their theoretical limits.^[^ ^41^, ^42^ ^]^ There are plenty of rooms for further device optimization through rationally tuning the BHJ morphology to suppress nonradiative recombination. In this regard, an ideal morphology should have many attributes, such as high crystallinity and favorable crystalline orientation of donor and acceptor for charge transport,^[^ ^23^, ^43^ ^]^ proper nanophase separation domain sizes for exciton dissociation,^[^ ^43^, ^44^ ^]^ and suitable vertical distribution of donor and acceptor for charge collection at the corresponding electrodes.^[^ ^32^, ^45^, ^46^, ^47^ ^]^ Although many efforts have been devoted to morphological optimization, systematic studies on these complex 3D multilength scale structural information are still very scarce.

Introducing additives is a common strategy to modify the BHJ morphology. For instance, liquid additives, 1,8‐diiodooctane (DIO)^[^ ^48^ ^]^ and 1‐chloronaphthalene (CN),^[^ ^49^ ^]^ were employed to effectively regulate the crystallization rate and molecular packing motif of the donor and/or acceptor, which in turn improved the exciton dissociation and charge‐transport properties of OSCs. However, the residual of these high‐boiling‐point additives would gradually evaporate from the active layer, thereby deteriorating the active layer morphology and the device performance.^[^ ^50^ ^]^ On the other hand, the development and utilization of volatile solid additives could overcome this drawback. The commercial volatile solid additives, 9,10‐anthracenedione (BDT‐1) and benzo[1,2‐*b*:4,5‐*b*′]dithiophene‐4,8‐dione (BDT‐2)^[^ ^51^ ^]^ have been used successfully to coordinate the molecular arrangement. The high‐crystallinity volatile solid additive dithieno[3,2‐*b*:2′,3′‐*d*]thiophene (DTT)^[^ ^52^ ^]^ can limit the excessive self‐assembly of nonfullerene acceptors, and then refine the phase separation and molecular packing. 1,4‐Diiodobenzene (DIB)^[^ ^53^ ^]^ has been reported to assist acceptor molecules to form a tighter molecular packing and more ordered microstructure.

**Figure 1:**
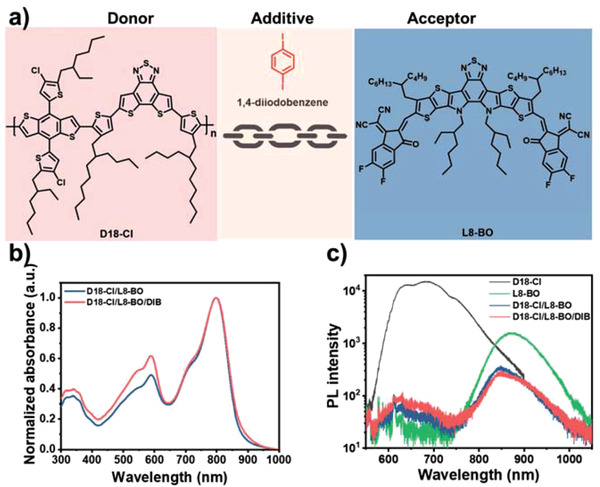
a) Chemical structures of D18‐Cl, DIB, and L8‐BO. b) UV–vis absorption spectra of blend films. c) PL spectra of the pure and blend films (excited at 532 nm).

Herein, we introduced the volatile solid additive DIB into a binary system comprised of a polymer donor, D18‐Cl,^[^ ^54^ ^]^ and a small‐molecule acceptor, L8‐BO.^[^ ^12^ ^]^ The chemical structures of D18‐Cl, L8‐BO, and DIB are shown in **Figure** **1**a. The previously reported HOMO/LUMO of D18‐Cl and L8‐BO are summarized in Figure S1 (Supporting Information).^[^ ^12^, ^54^ ^]^ The donor D18‐Cl has a deep HOMO energy level of −5.49 eV, whereas the acceptor, L8‐BO, as a Y6 derivative, adopts a relatively shallower LUMO energy level of −3.90 eV.^[^ ^15^ ^]^ Therefore, the binary D18‐Cl/L8‐BO system has a higher theoretical *V*_OC_ limit than conventional Y6‐based binary systems. As expected, the as‐cast D18‐Cl/L8‐BO device showed a *V*_OC_ of 0.950 V, higher than that of most previously reported high‐performance single‐junction OSCs. However, the device also exhibited a relatively lower *J*_SC_ of 24.1 mA cm^−2^ and an FF of 66.4%. Remarkably, with DIB, the OSCs showed significant improvements in *J*_SC_ and FF. The best‐performing cell demonstrated a PCE of 18.7% with a *V*_OC_ of 0.922 V, a *J*_SC_ of 26.6 mA cm^−2^, and an FF of 75.6%. To our knowledge, the *V*_OC_ achieved here is by far the highest among the reported OSCs with efficiencies over 17%. Density functional theory (DFT) calculations predicted that DIB interacts with the donor and acceptor through N–I interaction, which promoted the crystalline packing of donor and acceptor domains as well as the charge‐carrier mobility. Grazing‐incidence transmission small‐angle X‐ray scattering (GTSAXS) revealed that DIB induced the formation of smaller acceptor domains in the vertical direction, thus facilitating exciton dissociation. To sum up, our work here demonstrated the high competence of solid additives to tune the morphology, to obtain high *V*_OC_, *J*_SC_, and FF, which could be one of the most promising directions for the future device optimization of OSCs.

## 2 Results and Discussion

### 2.1 Optoelectronic Properties

The effect of DIB on the optoelectronic properties of the D18‐Cl/L8‐BO blend film was studied via ultraviolet–visible (UV–vis) absorption and photoluminescence (PL) spectroscopies. The blend films were fabricated on the basis of the optimized D/A mass ratio of 1/1.2. As shown in Figure 1b, there are two absorption peaks at 590 and 799 nm, corresponding to the maximum absorption of D18‐Cl and L8‐BO, respectively.^[^ ^12^, ^54^ ^]^ The D18‐Cl/L8‐BO blend film without or with 10 mg mL^−1^ DIB showed nearly identical absorption range; however, the presence of DIB enhanced the relative absorption in the D18‐Cl region, which might originate from the enhanced crystalline packing. Furthermore, the PL spectra (Figure 1c) of pure D18‐Cl and L8‐BO films on the glass substrate gave emission peaks at 685 and 873 nm, respectively. The PL signals of D18‐Cl and L8‐BO were significantly quenched in the D18‐Cl/L8‐BO and D18‐Cl/L8‐BO/DIB films, implying that there existed sufficient charge transfer between D18‐Cl and L8‐BO in both films.

### 2.2 Photovoltaic Properties

**Figure 2:**
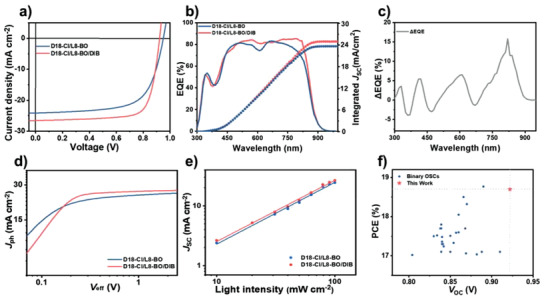
a) *J–V* curves. b) EQE response and integrated *J*_SC_ of best‐performing devices. c) ΔEQE characteristic. d) *J*_ph_ versus *V*_eff_ characteristics and e) *J*_SC_ versus light intensity of the optimized devices. f) Comparison of our results with previously reported PCE and *V*_OC_ for binary OSCs.

The conventional structure of patterned indium–tin oxide (ITO) glass/poly (3, 4‐ ethylenedioxythiophene): poly (styrenesulfonate) (PEDOT:PSS)/active layer/poly[(9,9‐bis(3′‐(N,N‐dimethylamino)propyl)‐2,7‐fluorene)‐alt‐5,5′‐bis(2,2′‐thiophene)‐2,6‐naphthalene‐1,4,5,8‐tetracaboxylic‐N,N′‐di(2‐ethylhexyl)imide] (PNDIT‐F3N)/Ag was adopted to fabricate the BHJ OSCs so as to investigate the photovoltaic device performance with/without the DIB additive. First, we optimized the D/A mass ratio and found that, at a ratio of 1/1.2, the as‐cast D18‐Cl/L8‐BO device reached the best PCE of 15.1% (Table S1, Supporting Information). Impressively, it exhibited a high *V*_OC_ of 0.950 V, confirming the potential of the binary D18‐Cl/L8‐BO system to deliver a high *V*_OC_. However, the *J*_SC_ and FF were not as high as those reported in over 17% efficiency Y6 systems, thereby leading to a lower PCE. Remarkably, the incorporation of DIB increased the PCE of the binary D18‐Cl/L8‐BO system substantially up to 17.9% (Table S3, Supporting Information). The thermal annealing treatment (90 ℃ for 10 min) further promoted the best PCE to 18.7% with a *V*_OC_ of 0.922 V, a *J*_SC_ of 26.6 mA cm^−2^, and an FF of 75.6%. To our knowledge, the *V*_OC_ achieved here is by far the highest among the reported OSCs with efficiencies over 17% (**Figure** **2**f; refs. 7–30 in the Supporting Information). The corresponding *J*–*V* curves are shown in Figure 2a. The device statistics for different DIB concentrations are summarized in Table S3 (Supporting Information). The external quantum efficiency (EQE) spectra of the representative D18‐Cl/L8‐BO‐ and D18‐Cl/L8‐BO/DIB‐based devices are presented in Figure 2b. Although both devices presented almost identical wavelength range, the EQE of D18‐Cl/L8‐BO/DIB‐based device displayed considerable improvements in the ranges of 350–450, 450–650, and 650–950 nm (Figure 2c), consistent with the trend of measured *J*_SC_ (**Table** **1**).

**Table 1:** Performance of the optimized binary OSCs under illumination of AM 1.5 G, 100 mW cm^−2^

Blend^a)^	*V*_OC_ ^b)^	*J*_SC_ ^b)^	FF^b)^	PCE^b)^	calculated *J*_SC_
	[V]	[mA cm^−2^]	[%]	[%]	[mA cm^−2^]
D18‐Cl/L8‐BO	0.950 (0.949 ± 0.002)	24.1 (23.8 ± 0.4)	66.4 (65.7 ± 0.9)	15.1 (14.8 ± 0.3)	23.7
D18‐Cl/L8‐BO/DIB ^c)^	0.922 (0.920 ± 0.003)	26.6 (26.1 ± 0.7)	75.6 (75.1 ± 0.5)	18.7 (18.3 ± 0.5)	25.0

^a)^ D/A = 1/1.2 (w/w);

^b)^ Average values with standard deviation (in parenthesis) are obtained from 15 independent devices;

^c)^ DIB concentration is 10 mg mL^−1^.

In addition, exciton dissociation and charge collection efficiency of the D18‐Cl/L8‐BO devices without and with the DIB additive were investigated by measuring the photocurrent density (*J*_ph_) dependence on the effective voltage (*V*_eff_), as shown in Figure 2d. The corresponding parameters are listed in Table S4 (Supporting Information). Here, *J*_ph_ is the difference between the current density under dark and the current density under 1 sun illumination (AM 1.5 G, 100 mW cm^−2^); *V*_eff_ was calculated by subtracting the voltage at zero photocurrent density with the externally applied voltage bias; *J*_sat_ is the saturation photocurrent density when *V*_eff_ is sufficiently high (>2.0 V); *J*_ph_ *^a^* and *J*_ph_ *^b^* are the photocurrent density under short‐circuit and maximum power output conditions, respectively. The exciton dissociation efficiency (*η*_diss_) was calculated by the ratio between *J*_ph_ *^a^* and *J*_sat_, while the charge collection efficiency (*η*_coll_) by the ratio between *J*_ph_ *^b^* and *J*_sat_. Hence, the *η*_diss_ and *η*_coll_ of the as‐cast D18‐Cl/L8‐BO OSCs are 94.9% and 82.3%, respectively, whereas the corresponding values are 98.5% and 85.2% for D18‐Cl/L8‐BO/DIB OSCs. It is evident that the DIB‐processed device had significantly enhanced exciton dissociation and charge collection ability, agreeing well with the observed higher *J*_SC_ and FF.

Besides, we also measured *J*_SC_ under different illumination light intensities (*P*_light_) in order to understand the dominant recombination mechanisms in D18‐Cl/L8‐BO‐based and D18‐Cl/L8‐BO/DIB‐based devices (Figure 2e). The *J*_SC_ values were fitted with *P*_light_ to the power of *α*,^[^ ^55^ ^]^ and the obtained exponents *α* are 0.995 and 0.996, respectively, suggesting that both systems did not suffer from severe bimolecular recombination. Moreover, the hole (*μ*_h_) and electron (*μ*_e_) mobilities of the as‐cast and DIB‐processed D18‐Cl/L8‐BO blend films were estimated by space charge limited current (SCLC) measurements (Figure S2 and Table S5, Supporting Information). The results showed that the best *μ*_h_ and *μ*_e_ for the as‐cast D18‐Cl/L8‐BO blend film are 11.1 × 10^−4^ and 5.5 × 10^−4^ cm^2^ V^−1^ s^−1^, while the corresponding mobilities for the D18‐Cl/L8‐BO/DIB film are 33.5 × 10^−4^ and 12.3 × 10^−4^ cm^2^ V^−1^ s^−1^. The increase in *μ*_h_ and *μ*_e_ for D18‐Cl/L8‐BO/DIB film is consistent with the observed more ordered crystalline packing of the donor and acceptor shown in the following section. Finally, the photostability of the as‐cast and DIB‐processed D18‐Cl/L8‐BO OSCs was investigated. The as‐cast and DIB‐processed devices still maintained 89% and 88% of their initial efficiency after 24 h of uninterrupted illumination, respectively (Figure S3, Supporting Information), indicating that the DIB processing does not affect the photostability of the OSCs, which is consistent with previous reports.^[^ ^50^ ^]^

### 2.3 Film Morphology

To understand the distinct performance of D18‐Cl/L8‐BO devices without/with DIB additive, the active layer morphology, both surface and bulk, was systematically characterized via atomic force microscopy (AFM) and grazing‐incidence X‐ray scattering techniques. The AFM images of D18‐Cl/L8‐BO blend films without/with the DIB additive are shown in Figure S4 (Supporting Information). The root‐mean‐square (RMS) roughnesses of the two films are similar, measured to be 1.10 and 1.25 nm, respectively, indicating smooth surfaces for good contact with the top electrode. On the other hand, the D18‐Cl/L8‐BO film exhibited a fibrous surface, while the D18‐Cl/L8‐BO/DIB film showed a granular surface, implying different D/A phase separation degrees, which were further confirmed by the following small‐angle X‐ray scattering results.

**Figure 3:**
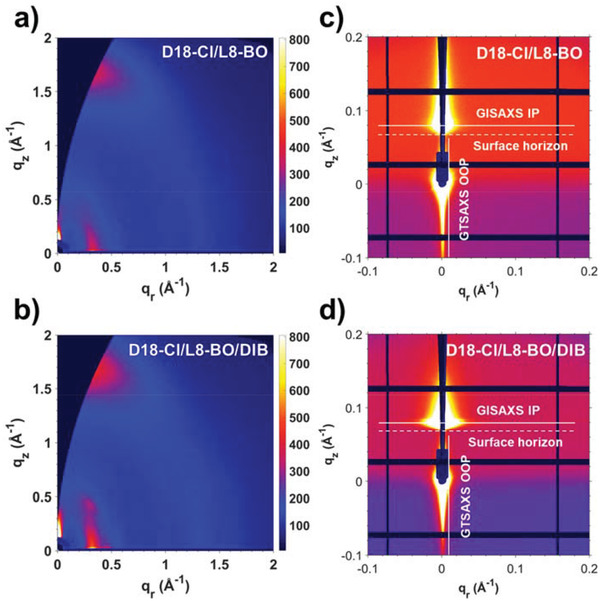
a,b) 2D GIWAXS and c,d) GTSAXS patterns of D18‐Cl/L8‐BO and D18‐Cl/L8‐BO/DIB blend films.

The molecular‐level bulk morphology was characterized by grazing‐incidence wide‐angle X‐ray scattering (GIWAXS). The 2D GIWAXS patterns of pure D18‐Cl and L8‐BO, and the corresponding intensity profiles along the in‐plane (IP) and out‐of‐plane (OOP) directions are shown in Figure S5a–c (Supporting Information). The pure D18‐Cl displayed a weak crystallinity with preferential face‐on orientation as the lamellar peak showed up at *q*_r_ = 0.320 Å^−1^ (*d* = 19.6 Å) and the *π*–*π* peak at *q_z_* = 1.67 Å^−1^ (*d* = 3.76 Å). The pure L8‐BO showed a higher face‐on order with a stronger *π*–*π* peak at *q_z_* = 1.73 Å^−1^ (*d* = 3.63 Å). In the small *q* region, the peak at (*q*_r_, *q_z_*) = (0.430, 0) Å^−1^ was assigned to the lamellar stacking, whereas the peak at (*q*_r_, *q_z_*) = (0.257, 0.435) Å^−1^ was probably due to the tilted end‐group stacking. As presented in **Figure** **3**, the GIWAXS pattern of the D18‐Cl/L8‐BO blend film without DIB resembled that of pure D18‐Cl film, but with stronger intensity, suggesting that the crystalline packing of D18‐Cl was improved in the blend film, but the packing of L8‐BO was weakened instead. In contrast, in the GIWAXS pattern of the D18‐Cl/L8‐BO blend film with DIB, the scattering signals from both D18‐Cl and L8‐BO can be clearly identified, demonstrating that the presence of DIB can enhance the crystallinity of both the donor and acceptor, in consistence with the observed higher electron and hole mobilities. Table S6 (Supporting Information) summarizes the peak analysis parameters of the blend films in IP and OOP directions.

The 3D nanoscale morphology of the blend films without and with DIB was then investigated by GTSAXS^[^ ^56^ ^]^ (Figure 3c,d). When the incident X‐rays were impinged on the front edge of the thin film cleaved at the center of the substrate, at a grazing angle of 0.6° with respect to the substrate, we could obtain the reflected scattering signals above the surface horizon and the transmitted signals below the surface horizon simultaneously. The former was used to perform horizontal linecuts to probe the IP domain size and the latter to perform vertical linecuts to probe the OOP domain size. Intensity profiles were fitted by the simple Born approximation with the Debye–Anderson–Brumberger (DAB) model and the fractal‐like network model.^[^ ^21^, ^57^ ^]^ The fitted domain sizes of the D18‐Cl/L8‐BO film are 25 and 27 nm in IP and OOP directions, respectively, and the corresponding values of the D18‐Cl/L8‐BO/DIB film are 22 and 14 nm, as illustrated in Figure S5d,e (Supporting Information). The domain sizes in the D18‐Cl/L8‐BO/DIB film shrunk toward the theoretical exciton diffusion length,^[^ ^58^ ^]^ particularly in the OOP directions (Figure S6, Supporting Information), which could contribute critically to the observed more efficient exciton dissociation and reduced charge recombination. Combining GIWAXS and GTSAXS results, it can be inferred that the appropriate enhancement of crystallinity to the donor and acceptor can induce the regulation of domain sizes in the vertical direction, thus favoring the *J*_SC_ and FF of the OSCs, which are also consistent with previous reports.^[^ ^59^ ^]^

### 2.4 DFT Calculation

**Figure 4:**
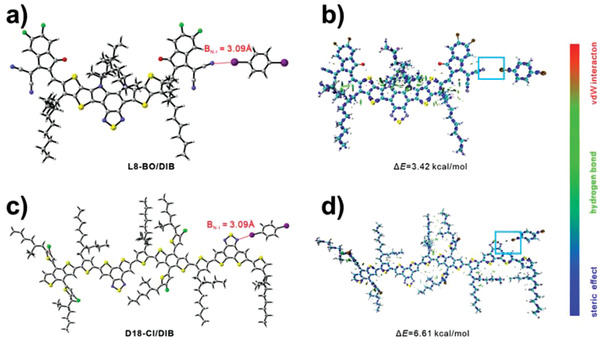
a,c) Optimized structures and b,d) isosurface maps of IRI for L8‐BO/DIB and D18‐Cl/DIB complexes at B3LYP/6‐31G(d) level.

The electrostatic potential surface (EPS) distributions of the donor, acceptor, and additive were analyzed to confirm the binding sites of D18‐Cl or L8‐BO with DIB. The EPS distributions were mapped on their van der Waals (vdW) surfaces (electron density isosurfaces of 0.001 au) (Figure S7, Supporting Information). The maximum negative and positive EPS values of L8‐BO are −39.88 and 39.08 kcal mol^−1^, and are distributed on the end groups and conjugated backbone, respectively. The hydrogen atom of terminal thiophene and the nitrogen atom of dithieno[3″,2″:3,4;2″″3″″:5,6]benzo[1,2‐*c*][1,2,5]thiadiazole in D18‐Cl result in the maximum positive (24.15 kcal mol^−1^) and negative (−30.21 kcal mol^−1^) EPS values, respectively. The EPS distributions of DIB exhibit that the maximum negative and positive EPS values are both located around iodine atoms. According to these EPS distributions, we optimized the L8‐BO/DIB and D18‐Cl/DIB complexes in the ground state (**Figure** **4**a,c). The L8‐BO or D18‐Cl combines with DIB via the interaction of N—I bonds with a bond length of 3.09 Å. Since there are two iodine atoms on DIB, it can bond L8‐BO and D18‐Cl molecules and maintain a stable D/A ratio during the film‐formation process. It may also mitigate the phase separation between L8‐BO and D18‐Cl, resulting in smaller domains (as observed by GTSAXS).

Then, we employed interaction region indicator (IRI) real space function^[^ ^60^ ^]^ to further study the interaction in L8‐BO/DIB and D18‐Cl/DIB complexes, which reveals the chemical bonding and weak interaction regions equally well. A stronger van der Waals interaction was found between D18‐Cl/DIB (marked zone) than L8‐BO/DIB (Figure 4b,d). Besides, the calculated interaction energy (Δ*E* = 6.61 kcal mol^−1^) between D18‐Cl and DIB was also larger than that of L8‐BO and DIB (Δ*E* = 3.42 kcal mol^−1^). These results indicated that DIB preferred to bond with D18‐Cl, which may be the origin of enhanced light absorption intensity of D18‐Cl in the DIB‐processed blend film.

### 2.5 Charge‐Transfer Kinetics

**Figure 5:**
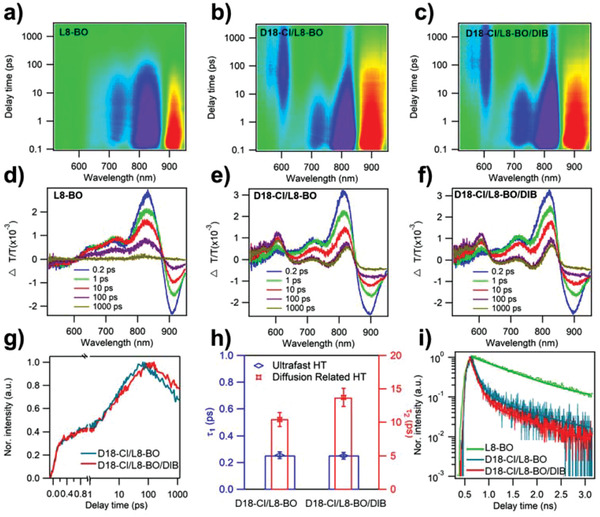
a–f) 2D color TA spectra and the representative spectra at indicated delay times of pure L8‐BO, D18‐2Cl/L8‐BO, and D18‐Cl/L8‐BO/DIB blend films under 750 nm excitation. g) TA kinetics and h) fitting results (*τ*_1_ and *τ*_2_) of relevant blends showing two steps hole‐transfer process. i) TRPL of pure and blend films under 750 nm excitation.

To further investigate the relationship between the EQE enhancements and the charge‐transfer process for D18‐Cl/L8‐BO and D18‐Cl/L8‐BO/DIB blend films, we employed femtosecond (fs) transient absorption (TA) spectroscopy to track the excited‐state dynamics. Here, we only excited excitons from the L8‐BO phase in different films by a pump laser of 750 nm and after a certain amount of delay time, probed the relative transmittance change (Δ*T*/*T*) with the white light continuum of 500–960 nm. The 2D color TA spectra of pure L8‐BO, D18‐Cl/L8‐BO, and D18‐Cl/L8‐BO/DIB films are shown in **Figure** **5**a–c, with representative TA spectra at several specific delay times plotted in Figure 5d–f for a clear comparison. The TA spectrum of the pure L8‐BO film (Figure 5a,d) exhibited a bleach peak at ≈820 nm and an absorption peak at ≈900 nm, corresponding to the combination of ground‐state bleach (GSB) and stimulated emission (SE), and excited‐state absorption (ESA) of L8‐BO excitons after photoexcitation, respectively. For D18‐Cl/L8‐BO blend film (Figure 5b,e), accompanied with the fast L8‐BO ESA signal (≈900 nm) decay, two new bleach peaks at 550–620 nm emerged within 200 ps, which were assigned to the D18‐Cl GSB signal, due to hole transfer from photoexcited L8‐BO to D18‐Cl. Similar spectral evolution and hole‐transfer process were also observed in D18‐Cl/L8‐BO/DIB blended film (Figure 5c,f).

As shown in Figure 5g, we chose the GSB kinetics at ≈605 nm of D18‐Cl to represent the hole‐transfer kinetics of blend films, since photoexcited pure L8‐BO film showed no signal at ≈605 nm. According to previous works,^[^ ^61^, ^62^ ^]^ the hole‐transfer kinetics can be fitted by a biexponential decay function (Table S7, Supporting Information) with an ultrafast interfacial hole‐transfer process (*τ*_1_) and a slow‐diffusion‐related transfer process (*τ*_2_). Figure 5h displays the comparison of *τ*_1_ and *τ*_2_ for two blends. D18‐Cl/L8‐BO and D18‐Cl/L8‐BO/DIB presented the similar ultrafast hole‐transfer process with *τ*_1_ = 0.255 ± 0.026 and 0.251 ± 0.025 ps. However, DIB‐processed blend film showed a slower L8‐BO exciton‐diffusion‐mediated hole‐transfer process with *τ*_2_ = 13.71 ± 1.37 ps, in contrast to that of as‐cast film (*τ*_2_ = 10.41 ± 1.04 ps). Although the domain size of L8‐BO was decreased after adding the DIB, the L8‐BO exciton‐diffusion‐mediated time extended in constant, probably due to the increase of L8‐BO exciton lifetime, which was supported by the morphology results that acceptor crystallinity was improved. Therefore, more L8‐BO excitons were capable of reaching the D/A interfaces for effective charge transfer and separation, leading to the observed stronger L8‐BO PL lifetime decay in blend with volatile solid additive DIB shown in time‐resolved photoluminescence (TRPL) spectra (Figure 5i). Besides the charge‐transfer process, the slower decay trend after 200 ps displayed in Figure 5g also suggested less recombination, in good agreement with the reduced recombination at the D/A interface owing to both donor and acceptor crystallinity improvement discussed before, and thus the enhancement of *J*_SC_ for DIB‐processed devices.

## 3 Conclusion

In this work, we combined a high LUMO acceptor L8‐BO and a low HOMO donor D18‐Cl to fabricate single‐junction binary OSCs. As expected, *V*_OC_ values higher than those reported in conventional PM6/Y6 devices were attained, yet with lower *J*_SC_ and FF. Fortunately, the incorporation of the volatile solid additive DIB could retain *J*_SC_ and FF by positively tuning the active layer morphology to facilitate exciton dissociation, charge transfer, transport, as well as collection. DFT calculations suggested that the two iodine atoms in DIB tend to bond with the nitrogen atoms on D18‐Cl and L8‐BO molecules, and modify their crystallization and phase separation kinetics. The weak bonding between the same donor or acceptor molecules may promote the crystallization of pure phase domains. On the other hand, the bonding between the donor and acceptor may reduce the phase separation domain sizes and retain the D/A ratio during film formation. To verify this hypothesis, we also fabricated the D18‐Cl/L8‐BO devices with another additive with two iodine atoms–DIO (Table S2, Supporting Information). The device performance with DIO was also improved, but not as high as with DIB, possibly because DIO is nonvolatile and would remain in the active layer. In summary, we demonstrated the high competence of solid additives with two iodine atoms to tune the BHJ morphology. It is worthwhile to systematically monitor the film growth kinetics of systems with solid additives with versatile functional groups in future studies.

## 4 Experimental Section

Details of device fabrication, measurement, and characterization can be found in the Supporting Information.

### Statistical Analysis

Statistical analyses were performed using Origin 2018. Data presentation and sample size were exhibited as the mean ± standard deviation (SD) in the corresponding table.

## Conflict of Interest

The authors declare no conflict of interest.

## Supporting information

Supporting Information

## Data Availability

The data that support the findings of this study are available from the corresponding author upon reasonable request.

## References

[1] G. Li , R. Zhu , Y. Yang , Nat. Photonics 2012, 6, 153.

[2] Y. Li , Acc. Chem. Res. 2012, 45, 723. 22288572 10.1021/ar2002446

[3] L. Lu , T. Zheng , Q. Wu , A. M. Schneider , D. Zhao , L. Yu , Chem. Rev. 2015, 115, 12666. 26252903 10.1021/acs.chemrev.5b00098

[4] C. Yan , S. Barlow , Z. Wang , H. Yan , A. K. Y. Jen , S. R. Marder , X. Zhan , Nat. Rev. Mater. 2018, 3, 18003.

[5] S. C. Price , A. C. Stuart , L. Yang , H. Zhou , W. You , J. Am. Chem. Soc. 2011, 133, 4625. 21375339 10.1021/ja1112595

[6] M. Zhang , X. Guo , W. Ma , H. Ade , J. Hou , Adv. Mater. 2015, 27, 4655. 26173152 10.1002/adma.201502110

[7] C. Sun , F. Pan , H. Bin , J. Zhang , L. Xue , B. Qiu , Z. Wei , Z. G. Zhang , Y. Li , Nat. Commun. 2018, 9, 743. 29467393 10.1038/s41467-018-03207-x PMC5821836

[8] Y. Lin , J. Wang , Z. G. Zhang , H. Bai , Y. Li , D. Zhu , X. Zhan , Adv. Mater. 2015, 27, 1170. 25580826 10.1002/adma.201404317

[9] G. Cai , P. Xue , Z. Chen , T. Li , K. Liu , W. Ma , J. Lian , P. Zeng , Y. Wang , R. P. S. Han , X. Zhan , Chem. Mater. 2019, 31, 6484.

[10] J. Yuan , Y. Zhang , L. Zhou , G. Zhang , H.‐L. Yip , T.‐K. Lau , X. Lu , C. Zhu , H. Peng , P. A. Johnson , M. Leclerc , Y. Cao , J. Ulanski , Y. Li , Y. Zou , Joule 2019, 3, 1140.

[11] G. Cai , W. Wang , J. Zhou , Y. Xiao , K. Liu , Z. Xie , X. Lu , J. Lian , P. Zeng , Y. Wang , X. Zhan , ACS Mater. Lett. 2019, 1, 367.

[12] C. Li , J. D. Zhou , J. L. Song , J. Q. Xu , H. T. Zhang , X. N. Zhang , J. Guo , L. Zhu , D. H. Wei , G. C. Han , J. Min , Y. Zhang , Z. Q. Xie , Y. P. Yi , H. Yan , F. Gao , F. Liu , Y. M. Sun , Nat. Energy 2021, 6, 605.

[13] J. Du , K. Hu , J. Zhang , L. Meng , J. Yue , I. Angunawela , H. Yan , S. Qin , X. Kong , Z. Zhang , B. Guan , H. Ade , Y. Li , Nat. Commun. 2021, 12, 5264. 34489439 10.1038/s41467-021-25638-9 PMC8421507

[14] G. Li , X. Zhang , L. O. Jones , J. M. Alzola , S. Mukherjee , L. W. Feng , W. Zhu , C. L. Stern , W. Huang , J. Yu , V. K. Sangwan , D. M. DeLongchamp , K. L. Kohlstedt , M. R. Wasielewski , M. C. Hersam , G. C. Schatz , A. Facchetti , T. J. Marks , J. Am. Chem. Soc. 2021, 143, 6123. 33848146 10.1021/jacs.1c00211

[15] J. Wang , X. Zhan , Acc. Chem. Res. 2021, 54, 132. 33284599 10.1021/acs.accounts.0c00575

[16] L. Meng , Y. Zhang , X. Wan , C. Li , X. Zhang , Y. Wang , X. Ke , Z. Xiao , L. Ding , R. Xia , H. L. Yip , Y. Cao , Y. Chen , Science 2018, 361, 1094. 30093603 10.1126/science.aat2612

[17] X. Xu , L. Yu , H. Yan , R. Li , Q. Peng , Energy Environ. Sci. 2020, 13, 4381.

[18] Z. Wang , Z. Peng , Z. Xiao , D. Seyitliyev , K. Gundogdu , L. Ding , H. Ade , Adv. Mater. 2020, 32, 2005386. 10.1002/adma.202005386 33150672

[19] L. Arunagiri , Z. Peng , X. Zou , H. Yu , G. Zhang , Z. Wang , J. Y. Lin Lai , J. Zhang , Y. Zheng , C. Cui , F. Huang , Y. Zou , K. S. Wong , P. C. Y. Chow , H. Ade , H. Yan , Joule 2020, 4, 1790.

[20] R. J. Ma , T. Liu , Z. H. Luo , K. Gao , K. Chen , G. Y. Zhang , W. Gao , Y. Q. Xiao , T. K. Lau , Q. P. Fan , Y. Z. Chen , L. K. Ma , H. L. Sun , G. L. Cai , T. Yang , X. H. Lu , E. G. Wang , C. L. Yang , A. K. Y. Jen , H. Yan , ACS Energy Lett. 2020, 5, 2711.

[21] J. Mai , Y. Xiao , G. Zhou , J. Wang , J. Zhu , N. Zhao , X. Zhan , X. Lu , Adv. Mater. 2018, 30, 1802888. 10.1002/adma.201802888 29978515

[22] Z. C. Zhou , S. J. Xu , J. N. Song , Y. Z. Jin , Q. H. Yue , Y. H. Qian , F. Liu , F. L. Zhang , X. Z. Zhu , Nat. Energy 2018, 3, 952.

[23] L. Zhu , M. Zhang , G. Q. Zhou , T. Y. Hao , J. Q. Xu , J. Wang , C. Q. Qiu , N. Prine , J. Ali , W. Feng , X. D. Gu , Z. F. Ma , Z. Tang , H. M. Zhu , L. Ying , Y. M. Zhang , F. Liu , Adv. Energy Mater. 2020, 10, 1904234.

[24] Y. Xiao , J. Yuan , G. Zhou , K. C. Ngan , X. Xia , J. Zhu , Y. Zou , N. Zhao , X. Zhan , X. Lu , J. Mater. Chem. A 2021, 9, 17030.

[25] G. Zhang , X. K. Chen , J. Xiao , P. C. Y. Chow , M. Ren , G. Kupgan , X. Jiao , C. C. S. Chan , X. Du , R. Xia , Z. Chen , J. Yuan , Y. Zhang , S. Zhang , Y. Liu , Y. Zou , H. Yan , K. S. Wong , V. Coropceanu , N. Li , C. J. Brabec , J. L. Bredas , H. L. Yip , Y. Cao , Nat. Commun. 2020, 11, 3943. 32770068 10.1038/s41467-020-17867-1 PMC7414148

[26] S. Liu , J. Yuan , W. Deng , M. Luo , Y. Xie , Q. Liang , Y. Zou , Z. He , H. Wu , Y. Cao , Nat. Photonics 2020, 14, 300.

[27] Y. Firdaus , V. M. Le Corre , S. Karuthedath , W. Liu , A. Markina , W. Huang , S. Chattopadhyay , M. M. Nahid , M. I. Nugraha , Y. Lin , A. Seitkhan , A. Basu , W. Zhang , I. McCulloch , H. Ade , J. Labram , F. Laquai , D. Andrienko , L. J. A. Koster , T. D. Anthopoulos , Nat. Commun. 2020, 11, 5220. 33060574 10.1038/s41467-020-19029-9 PMC7562871

[28] L. Ma , H. Yao , J. Wang , Y. Xu , M. Gao , Y. Zu , Y. Cui , S. Zhang , L. Ye , J. Hou , Angew. Chem., Int. Ed. Engl. 2021, 60, 15988. 33932274 10.1002/anie.202102622

[29] T. Wang , J. L. Bredas , J. Am. Chem. Soc. 2021, 143, 1822. 33492129 10.1021/jacs.0c09542

[30] A. J. Gillett , A. Privitera , R. Dilmurat , A. Karki , D. Qian , A. Pershin , G. Londi , W. K. Myers , J. Lee , J. Yuan , S.‐J. Ko , M. K. Riede , F. Gao , G. C. Bazan , A. Rao , T.‐Q. Nguyen , D. Beljonne , R. H. Friend , Nature 2021, 597, 666. 34588666 10.1038/s41586-021-03840-5

[31] M. Zhang , L. Zhu , G. Zhou , T. Hao , C. Qiu , Z. Zhao , Q. Hu , B. W. Larson , H. Zhu , Z. Ma , Z. Tang , W. Feng , Y. Zhang , T. P. Russell , F. Liu , Nat. Commun. 2021, 12, 309. 33436638 10.1038/s41467-020-20580-8 PMC7803987

[32] L. Zhan , S. Li , X. Xia , Y. Li , X. Lu , L. Zuo , M. Shi , H. Chen , Adv. Mater. 2021, 33, 2007231. 10.1002/adma.202007231 33598972

[33] H. Meng , C. Liao , M. Deng , X. Xu , L. Yu , Q. Peng , Angew. Chem., Int. Ed. Engl. 2021, 60, 22554. 34418267 10.1002/anie.202110550

[34] L. Liu , S. Chen , Y. Qu , X. Gao , L. Han , Z. Lin , L. Yang , W. Wang , N. Zheng , Y. Liang , Y. Tan , H. Xia , F. He , Adv. Mater. 2021, 33, 2101279. 10.1002/adma.202101279 34117664

[35] F. Liu , L. Zhou , W. Liu , Z. Zhou , Q. Yue , W. Zheng , R. Sun , W. Liu , S. Xu , H. Fan , L. Feng , Y. Yi , W. Zhang , X. Zhu , Adv. Mater. 2021, 33, 2100830. 10.1002/adma.202100830 34048104

[36] L. Hong , H. Yao , Y. Cui , P. Bi , T. Zhang , Y. Cheng , Y. Zu , J. Qin , R. Yu , Z. Ge , J. Hou , Adv. Mater. 2021, 33, 2103091. 10.1002/adma.202103091 34510580

[37] Z. Chen , W. Song , K. Yu , J. Ge , J. Zhang , L. Xie , R. Peng , Z. Ge , Joule 2021, 5, 2395.

[38] P. Bi , S. Zhang , Z. Chen , Y. Xu , Y. Cui , T. Zhang , J. Ren , J. Qin , L. Hong , X. Hao , J. Hou , Joule 2021, 5, 2408.

[39] Q. Liu , Y. Jiang , K. Jin , J. Qin , J. Xu , W. Li , J. Xiong , J. Liu , Z. Xiao , K. Sun , S. Yang , X. Zhang , L. Ding , Sci. Bull. 2020, 65, 272. 10.1016/j.scib.2020.01.001 36659090

[40] Y. Cui , Y. Xu , H. Yao , P. Bi , L. Hong , J. Zhang , Y. Zu , T. Zhang , J. Qin , J. Ren , Z. Chen , C. He , X. Hao , Z. Wei , J. Hou , Adv. Mater. 2021, 33, 2102420.

[41] J. Benduhn , K. Tvingstedt , F. Piersimoni , S. Ullbrich , Y. Fan , M. Tropiano , K. A. McGarry , O. Zeika , M. K. Riede , C. J. Douglas , S. Barlow , S. R. Marder , D. Neher , D. Spoltore , K. Vandewal , Nat. Energy 2017, 2, 17053.

[42] L. Ma , S. Zhang , J. Wang , Y. Xu , J. Hou , Chem. Commun. 2020, 56, 14337. 10.1039/d0cc05528j 33118555

[43] L. Zhang , X. Xu , B. Lin , H. Zhao , T. Li , J. Xin , Z. Bi , G. Qiu , S. Guo , K. Zhou , X. Zhan , W. Ma , Adv. Mater. 2018, 30, 1805041. 10.1002/adma.201805041 30368963

[44] Y. Zhang , M. T. Sajjad , O. Blaszczyk , A. J. Parnell , A. Ruseckas , L. A. Serrano , G. Cooke , I. D. W. Samuel , Chem. Mater. 2019, 31, 6548.

[45] X. Zhang , G. Li , S. Mukherjee , W. Huang , D. Zheng , L. W. Feng , Y. Chen , J. Wu , V. K. Sangwan , M. C. Hersam , D. M. DeLongchamp , J. Yu , A. Facchetti , T. J. Marks , Adv. Energy Mater. 2022, 12, 2102172.

[46] Y. Zhang , K. Liu , J. Huang , X. Xia , J. Cao , G. Zhao , P. W. K. Fong , Y. Zhu , F. Yan , Y. Yang , X. Lu , G. Li , Nat. Commun. 2021, 12, 4815. 34376697 10.1038/s41467-021-25148-8 PMC8355148

[47] W. Y. Yang , W. Wang , Y. H. Wang , R. Sun , J. Guo , H. N. Li , M. M. Shi , J. Guo , Y. Wu , T. Wang , G. H. Lu , C. J. Brabec , Y. F. Li , J. Min , Joule 2021, 5, 1209.

[48] J. K. Lee , W. L. Ma , C. J. Brabec , J. Yuen , J. S. Moon , J. Y. Kim , K. Lee , G. C. Bazan , A. J. Heeger , J. Am. Chem. Soc. 2008, 130, 3619. 18288842 10.1021/ja710079w

[49] C. V. Hoven , X.‐D. Dang , R. C. Coffin , J. Peet , T.‐Q. Nguyen , G. C. Bazan , Adv. Mater. 2010, 22, E63. 20217801 10.1002/adma.200903677

[50] Y. P. Xie , H. S. Ryu , L. L. Han , Y. H. Cai , X. P. Duan , D. H. Wei , H. Y. Woo , Y. M. Sun , Sci. China: Chem. 2021, 64, 2161.

[51] Y. Zhang , Y. Cho , J. Lee , J. Oh , S.‐H. Kang , S. M. Lee , B. Lee , L. Zhong , B. Huang , S. Lee , J.‐W. Lee , B. J. Kim , Y. Li , C. Yang , J. Mater. Chem. A 2020, 8, 13049.

[52] S. Bao , H. Yang , H. Fan , J. Zhang , Z. Wei , C. Cui , Y. Li , Adv. Mater. 2021, 33, 2105301. 10.1002/adma.202105301 34850986

[53] J. H. Fu , H. Y. Chen , P. H. Huang , Q. Q. Yu , H. Tang , S. S. Chen , S. Jung , K. Sun , C. Yang , S. R. Lu , Z. P. Kan , Z. Y. Xiao , G. Li , Nano Energy 2021, 84, 105862.

[54] A. Zeng , X. Ma , M. Pan , Y. Chen , R. Ma , H. Zhao , J. Zhang , H. K. Kim , A. Shang , S. Luo , I. C. Angunawela , Y. Chang , Z. Qi , H. Sun , J. Y. L. Lai , H. Ade , W. Ma , F. Zhang , H. Yan , Adv. Funct. Mater. 2021, 31, 2102413.

[55] I. Riedel , J. Parisi , V. Dyakonov , L. Lutsen , D. Vanderzande , J. C. Hummelen , Adv. Funct. Mater. 2004, 14, 38.

[56] X. Xia , T. K. Lau , X. Guo , Y. Li , M. Qin , K. Liu , Z. Chen , X. Zhan , Y. Xiao , P. F. Chan , H. Liu , L. Xu , G. Cai , N. Li , H. Zhu , G. Li , Y. Zhu , T. Zhu , X. Zhan , X. L. Wang , X. Lu , Nat. Commun. 2021, 12, 6226. 34711821 10.1038/s41467-021-26510-6 PMC8553947

[57] J. Mai , T.‐K. Lau , J. Li , S.‐H. Peng , C.‐S. Hsu , U. S. Jeng , J. Zeng , N. Zhao , X. Xiao , X. Lu , Chem. Mater. 2016, 28, 6186.

[58] J. D. A. Lin , O. V. Mikhnenko , J. Chen , Z. Masri , A. Ruseckas , A. Mikhailovsky , R. P. Raab , J. Liu , P. W. M. Blom , M. A. Loi , C. J. García‐Cervera , I. D. W. Samuel , T.‐Q. Nguyen , Mater. Horiz. 2014, 1, 280.

[59] Z. Wang , K. Gao , Y. Kan , M. Zhang , C. Qiu , L. Zhu , Z. Zhao , X. Peng , W. Feng , Z. Qian , X. Gu , A. K. Jen , B. Z. Tang , Y. Cao , Y. Zhang , F. Liu , Nat. Commun. 2021, 12, 332. 33436619 10.1038/s41467-020-20515-3 PMC7804468

[60] T. Lu , Q. Chen , Chem.‐Methods 2021, 1, 231.

[61] Z. Chen , X. Chen , Z. Jia , G. Zhou , J. Xu , Y. Wu , X. Xia , X. Li , X. Zhang , C. Deng , Y. Zhang , X. Lu , W. Liu , C. Zhang , Y. Yang , H. Zhu , Joule 2021, 5, 1832.

[62] Z. Chen , X. Chen , B. Qiu , G. Zhou , Z. Jia , W. Tao , Y. Li , Y. M. Yang , H. Zhu , J. Phys. Chem. Lett. 2020, 11, 3226. 32259443 10.1021/acs.jpclett.0c00919

