# Supplementary material for: Pushing the Efficiency of High Open‐Circuit Voltage Binary Organic Solar Cells by Vertical Morphology Tuning
Source: Adv Sci (Weinh). 2022 Mar 21;9(14):2200578. doi: 10.1002/advs.202200578 (PMC9108622; doi:10.1002/advs.202200578)
Supplement: Supplementary file 1 — Supporting Information [file ADVS-9-2200578-s001.pdf]

ADVANCED SCIENCE
Open Access

Supporting Information

for *Adv. Sci.*, DOI 10.1002/advs.202200578

Pushing the Efficiency of High Open-Circuit Voltage Binary Organic Solar Cells by Vertical Morphology Tuning

Guilong Cai, Zeng Chen, Xinxin Xia, Yuhao Li, Jiayu Wang, Heng Liu, PingPing Sun, Chao Li, Ruijie Ma, Yaoqiang Zhou, Weijie Chi, Jianqi Zhang*, Haiming Zhu, Jianbin Xu, He Yan, Xiaowei Zhan and Xinhui Lu**

Supporting Information

Pushing the Efficiency of High Open-Circuit Voltage Binary Organic Solar Cells by Vertical Morphology Tuning

Guilong Cai, Zeng Chen, Xinxin Xia, Yuhao Li, Jiayu Wang, Heng Liu, PingPing Sun, Chao Li, Ruijie Ma, Yaoqiang Zhou, Weijie Chi, Jianqi Zhang*, Haiming Zhu, Jianbin Xu, He Yan, Xiaowei Zhan, and Xinhui Lu**

Materials

Unless stated otherwise, all the chemical reagents and solvents used were obtained commercially and were used without further purification. Chloroform (99.5%), 1,8-diiodooctane (DIO) (97.0%), 1-chloronaphthalene (CN) (97.0%) and 1,4-diiodobenzene (DIB) (99.0%) were purchased from Sigma-Aldrich. D18-Cl (M_n = 45 kg mol^{-1}, M_w/M_n = 2.5) and PNDIT-F3N were purchased from OptiFocus Ltd. L8-BO was synthesized by our previous procedure.[1]

Characterization

The absorption spectra of thin films were measured using a JASCO-570 spectrophotometer (JASCO. Inc., Japan) on quartz substrates. Photoluminescence (PL) spectra were measured at room temperature using a commercially available Raman/PL spectrometer (Horiba, Inc.) with 532 nm laser source. The active layer surface was characterized in ambient conditions via an atomic force microscope (AFM) (Bruker, Dimension Icon) using a platinum–iridium (Pt–Ir) coating tip (Bruker: SCM-PIC-V2;

k: 3 N/m; f_0: 75 kHz). The scan area and speed were 2.0 μm × 2.0 μm and 0.6 Hz.

Device fabrication. All the devices are based on a conventional sandwich structure, patterned ITO glass/PEDOT:PSS/active layer/ PNDIT-F3N/Ag. The ITO substrates were first scrubbed by detergent and then sonicated with deionized water, acetone and isopropanol subsequently, and dried in an oven. The glass substrates were treated by UV-ozone for 20 min before use. PEDOT:PSS (Heraeus Clevios P VP AI 4083) was spin-cast onto the ITO substrates at 4000 rpm for 30 s, and then dried at 120 °C for 20 min in air. The donor/acceptor blends (1/1.2 weight ratio, total concentration is 15 mg mL^{-1}) were dissolved in a chloroform solution of DIB and stirred overnight at room temperature in a nitrogen-filled glove box. The blend solution was spin-cast at 3000 rpm for 30 s. Active layers were annealed on a 90 °C hotplate for 10 minutes after being coated. A thin PNDIT-F3N layer (~5 nm) was coated on the active layer, followed by the deposition of Ag electrode (100 nm).

SCLC Measurements. Hole-only or electron-only devices were fabricated as follows: ITO/PEDOT:PSS/active layer/Au for holes and ITO/ZnO/active layer/Al for electrons. The mobility was extracted by fitting the *J*-*V* curves using space charge limited current (SCLC) method, which follows $J = (9/8)\mu\varepsilon_r\varepsilon_0 V^2\exp(0.89(V/E_0 d)^{0.5})/d^3$.

Here, J refers to the current density, μ is hole or electron mobility, ε_r is relative dielectric constant of the transport medium, which is equal to 3, ε_0 is the permittivity of free space (8.85×10^{-12} F m^{-1}), $V = V_{appl} - V_{bi}$, where V_{appl} is the applied voltage to the device, and V_{bi} is the built-in voltage due to the difference in work function of the two electrodes (for hole-only diodes, V_{bi} is 0.2 V; for electron-only diodes, V_{bi} is 0 V). E_0 is

characteristic field, d is the thickness of the active layer and was measured by KLA-Tencor Alpha-Step D-600 Stylus Profiler.

GIWAXS and GTSAXS measurements. GIWAXS measurements were accomplished with a Xeuss 2.0 SAXS/WAXS laboratory beamline using a Cu X-ray source (8.05 keV, 1.54 Å) and a Pilatus3R 300K detector. The incidence angle is 0.15°. GTSAXS measurements were performed at beamline BL19U2 of National Facility for Protein Science Shanghai (NFPS) at Shanghai Synchrotron Radiation Facility (SSRF). The wavelength of X-ray radiation was set as 1.03 Å. Scattered X-ray intensities were collected using a Pilatus 2M detector (DECTRIS Ltd).

GTSAXS profile modeling

The IP and OOP scattering intensity profiles are modeled as:

$$I(q) = A1\langle P(q,R,z)\rangle S(q,R) + A2\ DAB(q) + A3$$

Here, A1, A2, A3 are prefactors, the background signal is modeled as $DAB(q)$ with DAB model (*J. Appl. Phys.* **1957**, 28, 679-683):

$$DAB(q) = \frac{8\pi\xi_{DAB}^3}{(1+q^2\xi_{DAB}^2)^2}$$

where ξ_{DAB} is the correlation length of amorphous intermixing phases. $\langle P(q,R,z)\rangle$ is the averaged form factor. Here, we employ the spherical form factor $P(q,r)$ following Schulz distribution $f(r,R,z)$ with the mean radius R and radius distribution $z > -1$ (*Phys. Rev. A*, **1987**, 35, 2200):

$$P(q,r) = \left[\frac{3(\sin qr - qr\cos qr)}{(qr)^3}\right]^2$$

$$f(r,R,z) = \left(\frac{z+1}{R}\right)^{z+1} r^z \exp\left[-\left(\frac{z+1}{R}r\right)\right]\frac{1}{\Gamma(z+1)}$$

$$\langle P(q,R,z)\rangle = \int_0^\infty P(q,r)f(r,R,z)dr$$

where $\Gamma(z+1)$ is the Gamma function. $S(q,R)$ is the structure factor which may differ for different systems, here we chose the conventional fractal-like network model as the structure factor for the fitting of both IP and OOP profiles (*Energy Environ. Sci.* **2013**, 6, 1938-1948):

$$S_f(\mathbf{q},R,\xi) = 1 + \frac{\sin[(D-1)\tan^{-1}(\mathbf{q}\xi)]}{(\mathbf{q}R)^D}\frac{D\Gamma(D-1)}{[1+1/(\mathbf{q}\xi)^2]^{(D-1)/2}}$$

where ξ is the correlation length of the fractal-like network formed by the aggregation of primary particles with the mean radius *R*. Here, the primary particles correspond to small molecular crystallites and the correlation length corresponds to the pure phase domain. The fractal dimension *D* is fixed to 3 here. The domain size of pure phase is determined by 2*Rg*, where $Rg = [D(1+D)/2]^{1/2}\,\xi$ is the gyration radius of this fractal-like network.

DFT calculation. DFT calculations were adopted to reveal the interaction mechanisms. For simplicity, two repetitive units of the donor polymer were simulated. All geometry optimizations in the ground states were carried out with B3LYP functional[2] in combination with the Def2SVP basis set[3] in chloroform. The empirical dispersion correction (D3)[4] was considered in all calculations. Frequency analysis was performed to confirm that we had obtained stable structures on the potential energy surfaces. When the solvent effect (in chloroform) was applicable, it was accounted for using the solvation model based on the density (SMD) model[5]. All calculations were carried out with Gaussian 16 A[6]. The type of interaction can be estimated by different colours in IRI. The blue, green, and red denoted steric effect, hydrogen effect, and vdW interaction, respectively. The IRI was calculated with Multifwn_3.7 code.

Transient Absorption Spectroscopy (TAS) Measurement. For femtosecond transient absorption spectroscopy, the fundamental output from Yb:KGW laser (1030

nm, 220 fs Gaussian fit, 100 kHz, Light Conversion Ltd) was separated to two light beams. One was introduced to NOPA (ORPHEUS-N, Light Conversion Ltd) to produce a certain wavelength for pump beam (here we use 720 nm), the other was focused onto a YAG plate to generate white light continuum as probe beam. The pump and probe overlapped on the sample at a small angle less than 10°. The transmitted probe light from sample was collected by a linear CCD array. Then we obtained transient differential transmission signals by equation shown below:

$$\frac{\Delta T}{T}=\frac{T_{\text{pump-on}}\text{-}T_{\text{pump-off}}}{T_{\text{pump-off}}}$$

All the samples were measured in vacuum environments.

Time-Resolved Photoluminescence Spectroscopy (TRPL). The TRPL measurements were conducted on a home-build far-field microfluorescence system (Olympus, IX73 inverted microscope). TRPL decay kinetics were collected under 750 nm excitation, using a TCSPC module (PicoHarp 300) and a SPAD detector (IDQ, id100) with an instrument response function ~100 ps.

Supporting Figures

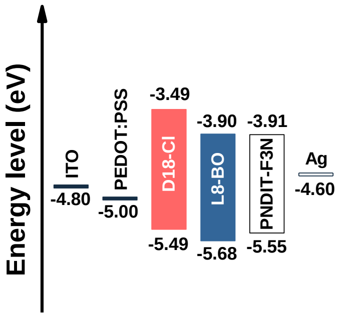

Figure S1. Energy-level alignment of materials.

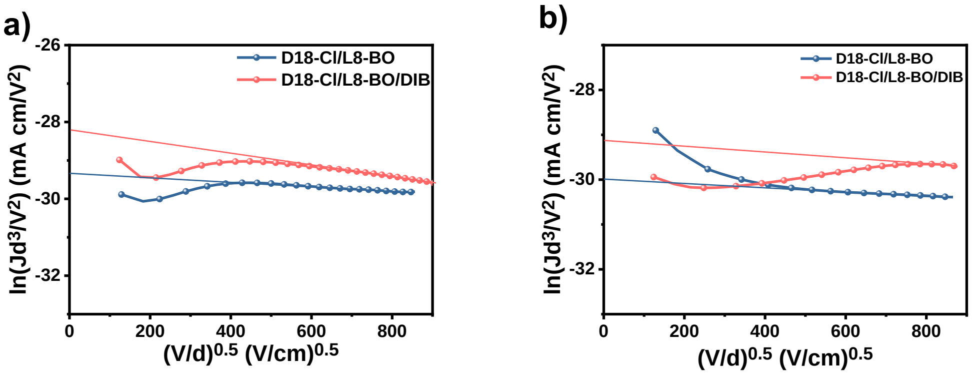

Figure S2. *J-V* characteristics in the dark for a) hole-only and b) electron-only devices based on optimal binary blends.

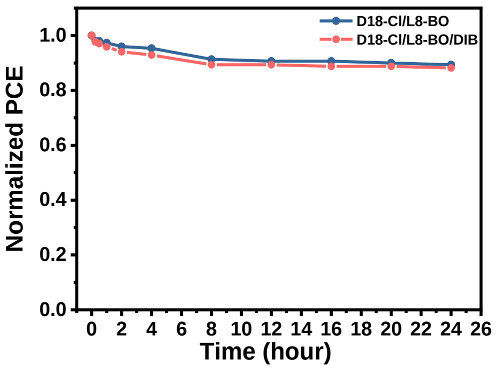

Figure S3. Photostability of as-cast and DIB-processed OSCs under the illumination of an AM 1.5 G solar simulator, 100 mW cm^{-2}.

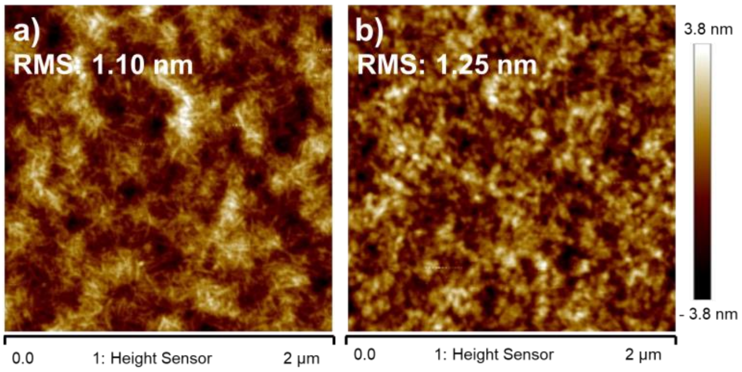

Figure S4. AFM height images of a) D18-Cl/L8-BO and b) D18-Cl/L8-BO/DIB blend films under optimal device conditions.

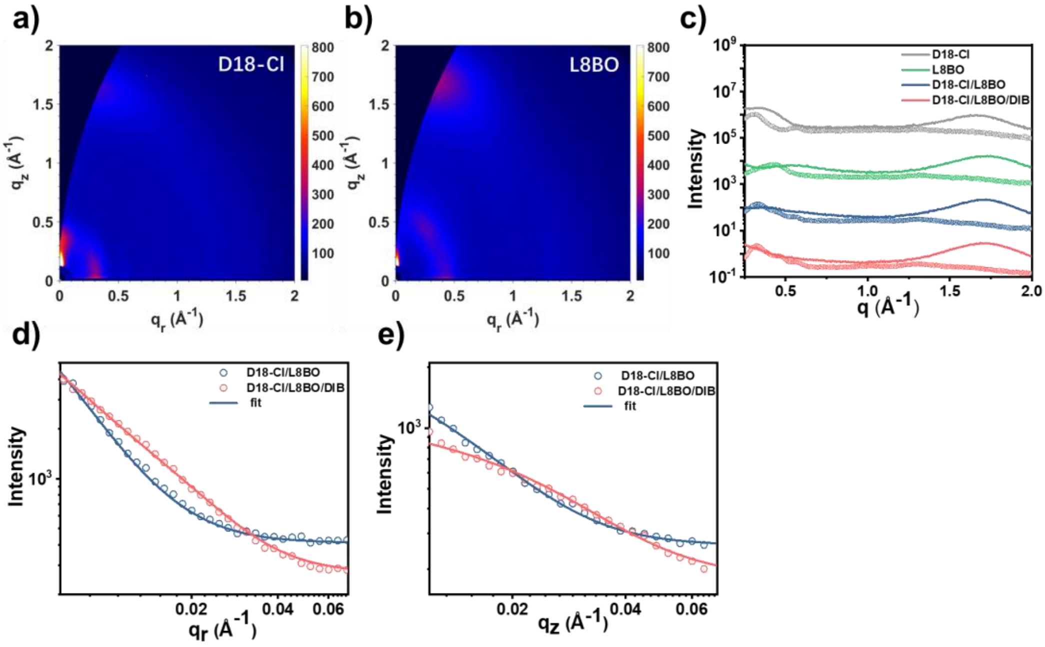

Figure S5. 2D GIWAXS patterns of a) D18-Cl, b) L8-BO and c) intensity profiles along the IP (dashed line) and OOP (solid line) directions of pure and blend films. d, e) The GTSAXS profiles and best fittings along the IP and OOP directions, respectively.

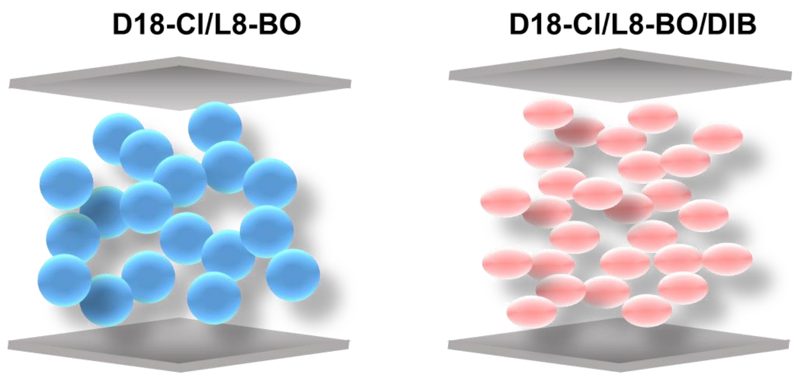

Figure S6. The diagram of acceptor phase in the D18-Cl/L8-BO and D18-Cl/L8-BO/DIB films.

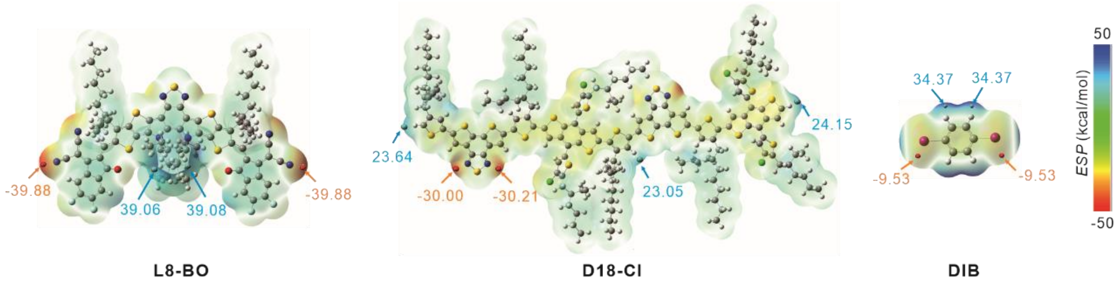

Figure S7. The electrostatic potential surfaces (EPS) distributions of L8-BO, D18-Cl and DIB at B3LYP/6-31G(d) level.

Supporting Tables

Table S1. Device data of as-cast OSCs based on D18-Cl/L8-BO with different D/A ratio.

D/A (w/w)	V_{OC}[a] (V)	J_{SC}[a] (mA cm^{-2})	FF[a] (%)	PCE[a] (%)
1/1	0.946 (0.944±0.003)	24.0 (24.2±0.3)	65.5 (64.2±1.6)	14.7 (14.4±0.4)
1/1.2	0.950 (0.949±0.002)	24.1 (23.8±0.4)	66.4 (65.7±0.9)	15.1 (14.8±0.3)
1/1.5	0.936 (0.934± 0.002)	24.3 (24.0±0.2)	65.7 (64.7±1.5)	14.9 (14.6±0.2)

[a] Average values (in parenthesis) are obtained from 15 independent devices.

Table S2. Device data of OSCs based on D18-Cl/L8-BO processed with different additives.

Additive	V_{OC} [a] (V)	J_{SC} [a] (mA cm^{-2})	FF [a] (%)	PCE [a] (%)
DIO (0.25%) [b]	0.920 (0.918±0.003)	25.5 (25.0±0.4)	70.9 (69.9±0.9)	16.6 (16.3±0.3)
CN (0.25%) [b]	0.918 (0.917±0.002)	25.0 (24.3±0.6)	69.6 (68.7±1.0)	15.8 (15.7±0.2)

[a] Average values (in parenthesis) are obtained from 15 independent devices. [b] Additive content (v/v) in chloroform.

Table S3. Device data of OSCs based on D18-Cl/L8-BO processed with different concentration of DIB solutions.

Concentration of DIB	V_{OC}[a] (V)	J_{SC}[a] (mA cm^{-2})	FF[a] (%)	PCE[a] (%)
10 mg mL^{-1}[b]	0.922<br>(0.921±0.002)	26.2<br>(25.7±0.5)	74.1<br>(73.4±1.1)	17.9<br>(17.5±0.4)
5 mg mL^{-1}[c]	0.924<br>(0.922±0.003)	25.5<br>(25.0±0.4)	72.5<br>(71.9±0.7)	17.0<br>(16.7±0.2)
10 mg mL^{-1}[c]	0.922<br>(0.920±0.003)	26.6<br>(26.1±0.7)	75.6<br>(75.1±0.5)	18.7<br>(18.3±0.5)
15 mg mL^{-1}[c]	0.910<br>(0.911±0.001)	24.6<br>(24.2±0.5)	73.1<br>(71.7±1.3)	16.3<br>(16.0±0.3)

[a] Average values (in parenthesis) are obtained from 15 independent devices. [b] Devices without any post-treatment. [c] Devices performed thermal annealing process at 90 °C for 10 min.

Table S4. The parameters of exciton dissociation efficiency and charge collection efficiency.

Sample	J_{sat} (mA cm^{-2})	J_{ph}[a] (mA cm^{-2})	J_{ph}[b] (mA cm^{-2})	η_{diss} (%)	η_{coll} (%)
D18-Cl/L8-BO	25.4	24.1	20.9	94.9	82.3
D18-Cl/L8-BO/DIB	27.0	26.6	23.0	98.5	85.2

[a] Under short circuit condition; [b] Under the maximal power output condition.

Table S5. Charge Mobilities of Blend Films Measured by SCLC Method.

Active layer	μ_h (× 10^{-4} cm^2 V^{-1} s^{-1}) [a]	μ_e (× 10^{-4} cm^2 V^{-1} s^{-1}) [a]
D18-Cl/L8-BO	11.1±0.5	5.5±0.8
D18-Cl/L8-BO/DIB	33.5±0.9	12.3±1.1

[a] Average values are obtained from 5 independent devices.

Table S6. Calculated GIWAXS characteristics of blend films.

Film	Direction	q ($Å^{-1}$)	d-spacing (Å)	FWHM ($Å^{-1}$)	CCL (Å)
D18-Cl/L8-BO	IP	0.326	19.0	0.087	65.0
D18-Cl/L8-BO/DIB	IP	0.322	19.5	0.082	68.9
		0.432	14.5	0.163	34.7
D18-Cl/L8-BO	OOP	1.72	3.65	0.295	19.2
D18-Cl/L8-BO/DIB	OOP	1.72	3.65	0.288	19.6

Table S7. Detailed kinetic fitting parameters of hole transfer for optimal blend films.

Blend	A_1 (%)	τ_1 (ps)	A_2 (%)	τ_2 (ps)
D18-Cl/L8-BO	38.5	0.255±0.026	61.5	10.41±1.04
D18-Cl/L8-BO/DIB	40.0	0.251±0.025	60.0	13.71±1.37

The hole transfer kinetics of blend films can be fitted by a biexponential function: $i = A_1 \exp(-t/\tau_1) + A_2 \exp(-t/\tau_2)$, with fast and slow lifetimes of τ_1 and τ_2 and prefactors of A_1 and A_2.

Table S8. Lifetime derived from TRPL measurements.

Blend	A_1 (%)	τ_1 (ns)	A_2 (%)	τ_2 (ns)
L8-BO	-	0.868	-	-
D18-Cl/L8-BO	88	0.111	12	0.894
D18-Cl/L8-BO/DIB	92	0.112	8	0.914

Supporting References

[1] C. Li, J. D. Zhou, J. L. Song, J. Q. Xu, H. T. Zhang, X. N. Zhang, J. Guo, L. Zhu, D. H. Wei, G. C. Han, J. Min, Y. Zhang, Z. Q. Xie, Y. P. Yi, H. Yan, F. Gao, F. Liu, Y. M. Sun, *Nat. Energy* **2021**, *6*, 605.

[2] A. D. Becke, *J. Chem. Phys.* **1992**, *97*, 9173.

[3] F. Weigend, R. Ahlrichs, *Phys. Chem. Chem. Phys.* **2005**, *7*, 3297.

[4] S. Grimme, J. Antony, S. Ehrlich, H. Krieg, *J. Chem. Phys.* **2010**, *132*, 154104.

[5] A. V. Marenich, C. J. Cramer, D. G. Truhlar, *J. Phys. Chem. B* **2009**, *113*, 6378.

[6] M. J. Frisch, G. W. Trucks, H. B. Schlegel, G. E. Scuseria, M. A. Robb, J. R. Cheeseman, G. Scalmani, V. Barone, G. A. Petersson, H. Nakatsuji, X. Li, M. Caricato, A. V. Marenich, J. Bloino, B. G. Janesko, R. Gomperts, B. Mennucci, H. P. Hratchian, J. V. Ortiz, A. F. Izmaylov, J. L. Sonnenberg, Williams, F. Ding, F. Lipparini, F. Egidi, J. Goings, B. Peng, A. Petrone, T. Henderson, D. Ranasinghe, V. G. Zakrzewski, J. Gao, N. Rega, G. Zheng, W. Liang, M. Hada, M. Ehara, K. Toyota, R. Fukuda, J. Hasegawa, M. Ishida, T. Nakajima, Y. Honda, O. Kitao, H. Nakai, T.

Vreven, K. Throssell, J. A. Montgomery Jr., J. E. Peralta, F. Ogliaro, M. J. Bearpark, J. J. Heyd, E. N. Brothers, K. N. Kudin, V. N. Staroverov, T. A. Keith, R. Kobayashi, J. Normand, K. Raghavachari, A. P. Rendell, J. C. Burant, S. S. Iyengar, J. Tomasi, M. Cossi, J. M. Millam, M. Klene, C. Adamo, R. Cammi, J. W. Ochterski, R. L. Martin, K. Morokuma, O. Farkas, J. B. Foresman, D. J. Fox, Wallingford, CT 2016.

[7] Y. Zhang, K. Liu, J. Huang, X. Xia, J. Cao, G. Zhao, P. W. K. Fong, Y. Zhu, F. Yan, Y. Yang, X. Lu, G. Li, *Nat. Commun.* **2021**, *12*, 4815.

[8] X. Yuan, Y. Zhao, T. Zhan, J. Oh, J. Zhou, J. Li, X. Wang, Z. Wang, S. Pang, P. Cai, C. Yang, Z. He, Z. Xie, C. Duan, F. Huang, Y. Cao, *Energy Environ. Sci.* **2021**, *14*, 5530.

[9] Q. Wu, H. Ning, Q. Jiang, P. Han, M. Lin, G. Zhang, J. Chen, H. Chen, S. Zeng, J. Gao, J. Liu, F. He, *Energy Environ. Sci.* **2021**, *14*, 5919.

[10] Y. Wei, J. Yu, L. Qin, H. Chen, X. Wu, Z. Wei, X. Zhang, Z. Xiao, L. Ding, F. Gao, H. Huang, *Energy Environ. Sci.* **2021**, *14*, 2314.

[11] J. L. Wang, C. Yang, Q. An, H. R. Bai, H. F. Zhi, H. S. Ryu, A. Mahmood, X. Zhao, S. Zhang, H. Y. Woo, J. Wang, *Angew. Chem. Int. Ed. Engl.* **2021**. *60*, 19241.

[12] L. Ma, H. Yao, J. Wang, Y. Xu, M. Gao, Y. Zu, Y. Cui, S. Zhang, L. Ye, J. Hou, *Angew. Chem. Int. Ed. Engl.* **2021**, *60*, 15988.

[13] J. Lv, H. Tang, J. Huang, C. Yan, K. Liu, Q. Yang, D. Hu, R. Singh, J. Lee, S. Lu, G. Li, Z. Kan, *Energy Environ. Sci.* **2021**, *14*, 3044.

[14] Q. Kang, Z. Zheng, Y. Zu, Q. Liao, P. Bi, S. Zhang, Y. Yang, B. Xu, J. Hou, *Joule* **2021**, *5*, 646.

[15] J. H. Fu, H. Y. Chen, P. H. Huang, Q. Q. Yu, H. Tang, S. S. Chen, S. Jung, K. Sun, C. Yang, S. R. Lu, Z. P. Kan, Z. Y. Xiao, G. Li, *Nano Energy* **2021**, *84*, 105862.

[16] Y. Chen, R. Ma, T. Liu, Y. Xiao, H. K. Kim, J. Zhang, C. Ma, H. Sun, F. Bai, X. Guo, K. S. Wong, X. Lu, H. Yan, *Adv. Energy Mater.* **2021**, *11*, 2003777.

[17] Y. Chang, J. Zhang, Y. Chen, G. Chai, X. Xu, L. Yu, R. Ma, H. Yu, T. Liu, P. Liu, Q. Peng, H. Yan, *Adv. Energy Mater.* **2021**, *11*, 2100079.

[18] Z. Zhang, Y. Li, G. Cai, Y. Zhang, X. Lu, Y. Lin, *J. Am. Chem. Soc.* **2020**, *142*, 18741.

[19] X. Xu, L. Yu, H. Yan, R. Li, Q. Peng, *Energy Environ. Sci.* **2020**, *13*, 4381.

[20] T. Wang, R. Sun, M. Shi, F. Pan, Z. Hu, F. Huang, Y. Li, J. Min, *Adv. Energy Mater.* **2020**, *10*, 2000590.

[21] F. Qi, K. Jiang, F. Lin, Z. Wu, H. Zhang, W. Gao, Y. Li, Z. Cai, H. Y. Woo, Z. Zhu, A. K. Y. Jen, *ACS Energy Lett.* **2020**, *6*, 9.

[22] R. Ma, T. Liu, Z. Luo, Q. Guo, Y. Xiao, Y. Chen, X. Li, S. Luo, X. Lu, M. Zhang, Y. Li, H. Yan, *Sci. China Chem.* **2020**, *63*, 325.

[23] Z. H. Luo, R. J. Ma, T. Liu, J. W. Yu, Y. Q. Xiao, R. Sun, G. S. Xie, J. Yuan, Y. Z. Chen, K. Chen, G. D. Chai, H. L. Sun, J. Min, J. Zhang, Y. P. Zou, C. L. Yang, X. H. Lu, F. Gao, H. Yan, *Joule* **2020**, *4*, 1236.

[24] X. Guo, Q. Fan, J. Wu, G. Li, Z. Peng, W. Su, J. Lin, L. Hou, Y. Qin, H. Ade, L. Ye, M. Zhang, Y. Li, *Angew. Chem. Int. Ed.* **2020**, *60*, 2322.

[25] W. Gao, H. Fu, Y. Li, F. Lin, R. Sun, Z. Wu, X. Wu, C. Zhong, J. Min, J. Luo, H. Y. Woo, Z. Zhu, A. K. Y. Jen, *Adv. Energy Mater.* **2020**, *11*, 2003177.

[26] T. Zhang, C. An, P. Bi, Q. Lv, J. Qin, L. Hong, Y. Cui, S. Zhang, J. Hou, *Adv. Energy Mater.* **2021**, *11*, 2101705.

[27] L. Liu, S. Chen, Y. Qu, X. Gao, L. Han, Z. Lin, L. Yang, W. Wang, N. Zheng, Y. Liang, Y. Tan, H. Xia, F. He, *Adv. Mater.* **2021**, *33*, 2101279.

[28] L. Hong, H. Yao, Y. Cui, P. Bi, T. Zhang, Y. Cheng, Y. Zu, J. Qin, R. Yu, Z. Ge, J. Hou, *Adv. Mater.* **2021**, 2103091.

[29] Y. Cui, H. Yao, J. Zhang, K. Xian, T. Zhang, L. Hong, Y. Wang, Y. Xu, K. Ma, C. An, C. He, Z. Wei, F. Gao, J. Hou, *Adv. Mater.* **2020**, *32*, 1908205.

[30] Y. Cui, Y. Xu, H. Yao, P. Bi, L. Hong, J. Zhang, Y. Zu, T. Zhang, J. Qin, J. Ren, Z. Chen, C. He, X. Hao, Z. Wei, J. Hou, *Adv. Mater.* **2021**, 2102420.
